# Processing Load Imposed by Line Breaks in English Temporal Wh-Questions

**DOI:** 10.3389/fpsyg.2016.01465

**Published:** 2016-10-07

**Authors:** Masako Hirotani, J. Michael Terry, Norihiro Sadato

**Affiliations:** ^1^Language and Brain Laboratory, School of Linguistics and Language Studies and Institute of Cognitive Science, Carleton UniversityOttawa, ON, Canada; ^2^Department of System Neuroscience, National Institute for Physiological SciencesOkazaki, Japan; ^3^Department of Linguistics, The University of North Carolina at Chapel HillChapel Hill, NC, USA

**Keywords:** implicit prosody, inner voice, line breaks, wh-questions, silent reading, written text, sentence processing, sentence comprehension

## Abstract

Prosody plays an important role in online sentence processing both explicitly and implicitly. It has been shown that prosodically packaging together parts of a sentence that are interpreted together facilitates processing of the sentence. This applies not only to explicit prosody but also implicit prosody. The present work hypothesizes that a line break in a written text induces an implicit prosodic break, which, in turn, should result in a processing bias for interpreting English wh-questions. Two experiments—one self-paced reading study and one questionnaire study—are reported. Both supported the “line break” hypothesis mentioned above. The results of the self-paced reading experiment showed that unambiguous wh-questions were read faster when the location of line breaks (or frame breaks) matched the scope of a wh-phrase (main or embedded clause) than when they did not. The questionnaire tested sentences with an ambiguous wh-phrase, one that could attach either to the main or the embedded clause. These sentences were interpreted as attaching to the main clause more often than to the embedded clause when a line break appeared after the main verb, but not when it appeared after the embedded verb.

## Introduction

Previous psycholinguistic research has established the importance of chunking utterances into prosodic units that are created in accordance with both grammatical (e.g., Cooper and Paccia-Copper, 1980; Selkirk, 1984; Nespor and Vogel, 1986; Jun, 2005; Ladd, 2009) and processing constraints (see e.g., Beckman, 1996; Frazier et al., 2006). These prosodic groups not only influence listeners' sentence processing in the spoken domain but also readers' sentence processing in the written domain, i.e., the effect of implicit prosody (e.g., Bader, 1998; Fodor, 2002). In this paper, we report the results of two experiments that test the hypothesis that line breaks inserted into written text induce implicit prosodic boundaries. It is argued that depending on their placement, these implicit boundaries help or hinder sentence processing.

It is well known that garden-path effects can be avoided by the use of prosodic phrasing (e.g., Price et al., 1991; Pynte and Prieur, 1996; Kjelgaard and Speer, 1999; Schafer et al., 2000; for an overview, see Cutler et al., 1997; Frazier et al., 2006; Speer and Blodgett, 2006). Such immediate use of prosodic phrasing has been demonstrated not only in offline studies but in both eye-tracking (e.g., Snedeker and Trueswell, 2003) and Event Related Potential (ERP) experiments as well (e.g., Steinhauer et al., 1999; Pannekamp et al., 2005; Eckstein and Friederici, 2006). In ERP studies, Closure Positive Shift (CPS), a positive wave that occurs at prosodic boundaries, was found (Steinhauer et al., 1999). CPS can help us determine whether or not a listener's processing of a sentence was influenced by the prosodic phrasing of a sentence presented earlier (e.g., Steinhauer et al., 1999; Wolff et al., 2008).

Various levels of prosodic phrasing have been proposed in a number of linguistic theories. In this paper, we assume the prosodic hierarchy proposed for English and other languages by Selkirk (1984), in which higher levels of a prosodic phrase exhaustively contain lower levels; each intonational phrase dominates one or more major phrases, each major phrase one or more minor phrases, and each minor phrase one or more prosodic words (see also Beckman and Pierrehumbert, 1986). Previous research in psycholinguistics argues that listeners make use of these different levels of prosodic phrases during sentence processing. It is suggested that intonational phrase boundaries are critical for assigning an interpretation to a sentence, whereas major phrases (also known as “immediate phrases”), for example, resolve the attachment ambiguity of phrases within a sentence (Schafer, 1997). Whereas the work just mentioned highlights the important functions of different levels of prosodic phrasing, it has also been shown that listeners constantly compute the relative sizes of prosodic phrasings within a sentence and use this information when they need to make parsing decisions during online sentence processing (Clifton et al., 2002). Furthermore, more recent evidence suggests that listeners take into account information regarding the choice of speakers made for prosodic phrasing when they process uttered sentences online (Clifton et al., 2006).

Although the use of prosodic phrasing may seem complicated (see above), the essential function of prosodic boundaries is straight forward: to bind elements within the same prosodic phrase and process them together (see Frazier et al., 2006). From the view point of processing load, the more prosodic units we can process and remove from our working memory, the less costly the entire process is. In other words, it is less costly if we have fewer prosodic phrases to hold onto in our working memory. It has also been argued that the insertion of a prosodic boundary does the job of separating previously processed materials from upcoming ones (see Frazier et al., 2004; Watson and Gibson, 2004, 2005; Hirotani, 2005). This might not necessarily lead to reducing the processing load involved. However, it might help listeners clarify the structure of a sentence they are processing (For the use of prosodic phrasing in production, see e.g., Schafer et al., 2000; Watson and Gibson, 2005).

As noted earlier, the effect of prosody is not confined to the spoken domain. During the silent reading of sentences, readers are influenced by prosodic boundaries inserted implicitly (e.g., Bader, 1998; Fodor, 2002; Hirose, 2003). Various reading studies have shown that across languages with similar sentence structures (e.g., English, Spanish), differences in the preferred attachment sites for ambiguous relative clauses can be explained by the differences in the surface realization of prosodic phrasing in those languages (Fodor, 2002). The effect of CPS found in auditory ERP experiments was also observed in a reading study (Steinhauer and Friederici, 2001). Furthermore, it has been shown that punctuation in text (e.g., comma, period) and prosodic features of words influence reading times of sentences in eye-tracking experiments (e.g., Ashby and Rayner, 2004; Ashby and Clifton, 2005; Hirotani et al., 2006).

### Present study

The purpose of the two studies reported herein was to investigate whether or not packaging parts of wh-questions into “implicit” prosodic units facilitates sentence processing. The experiments tested wh-questions like those presented in (1) (see Straub et al., 2001 for production studies on wh-questions). The wh-question in (1a) requires a main clause interpretation in which the wh-fragment, “when tomorrow,” must be interpreted as part of the main clause. This is because the tense of the wh-fragment is compatible with the main verb, but not the verb in the embedded clause. In contrast, “when tomorrow” in the wh-question (1b) must be interpreted as part of the embedded clause. This is because the future tensed wh-fragment can only be interpreted as part of the future tensed embedded clause.

(1) a. Main Clause Interpretation
  When tomorrow_i_ will Susie learn t_i_ that Bill made an important phone call?
b. Embedded Clause Interpretation
  When tomorrow_j_ did Susie learn that Bill will make an important phone call t_j_?

We hypothesize that grouping the materials that can be interpreted together into the same prosodic unit facilitates the processing of the sentence and as a result induces less processing load. In addition, we hypothesize that frame breaks in self-paced reading studies or line breaks in text induce implicit prosodic boundaries. Based on these hypotheses, the implicit prosodic packaging illustrated in (2a) in which the future tensed wh-fragment and the future tensed main clause are packaged together should be less costly than the prosodic packaging illustrated in (2b) in which the future tensed wh-fragment and the past tensed main clause are packaged together. The elements of the first prosodic unit in (2a) can be processed together, whereas the elements of the first prosodic unit in (2b) cannot. During silent reading, we expect the wh-question in (2a) to be read faster than the wh-question in (2b). Our hypothesis that the effect of prosodic packaging works the same even when the sentences have implicit prosodic boundaries is based on previous research (see above). What is new in the present study is the hypothesis that both frame breaks in self-paced reading and line breaks in text induce implicit prosodic boundaries.

(2) Parentheses below and hereafter indicate implicit prosodic boundaries.
a. (When tomorrow_i_ will Susie learn t_i_) (that Bill made an important phone call)?
b. (When tomorrow_j_ did Susie learn) (that Bill will make an important phone call t_j_)?

Some readers might wonder if the effect described above, i.e., (2a) being less costly than (2b), might be due to Active Filler Strategy (Frazier and Flores d'Arcais, 1989) or Minimal Chain Principle (De Vincenzi, 1991), or perhaps some other general processing principle requiring parts of a sentence to be processed as soon as possible. The Active Filler Strategy postulates a gap (in this case the trace of a wh-phrase) at the earliest syntactically legal position after encountering a filler (here a wh-phrase). Thus, it predicts the trace inside the main clause in (2a). The Minimal Chain Principle ensures that any chain of empty categories within a sentence contain as few links (here traces) as possible. Following this principle, in postulating a trace inside the main clause in the above examples is less costly than positing a trace inside the embedded clause. For the example sentences in (1), the processing preference for the wh-phrase is predicted to be the same by either the Active Filler Strategy or the Minimal Chain Principle, i.e., the wh-phrase is preferred to be interpreted as part of the main clause, as in (1a), to the embedded clause, as in (1b). In fact, processing the temporal wh-phrase within the main clause is impossible for (1b) due to the tense clash between “when tomorrow” and the main clause (“did”). Both the Active Filler Strategy and the Minimal Chain Principle are capable of explaining the effect outlined in (1) (For the work concerning processing wh-questions in general, see Aoshima et al., 2004.) Therefore, to argue that the effect is one of implicit prosodic packaging, it is important to show it to be independent from other processing principles.

In one of the reported experiments (Experiment 1), different patterns of implicit prosody for the sentences in (1), induced by frame breaks in a self-paced reading study, will be looked at, as well as those in (2).

(3) a. (When tomorrow_i_) (will Susie learn t_i_ that Bill made an important phone call)?
b. (When tomorrow_j_) (did Susie learn that Bill will make an important phone call t_j_)?

For (3a), above, assume that either the Active Filler Strategy or the Minimal Chain Principle is in effect. For example, when we read “when” (a filler), it is predicated that we postulate a gap (a trace of the wh-phrase). However, the “implicit prosodic packaging” of “when tomorrow” will not work, as we cannot assign an interpretation to it. The prosodic pattern indicated above might work better for (3b), as it is not possible to interpret “when tomorrow” as modifying the main clause due to the tense clash (“did”), as mentioned already.

As noted above, it is impossible to assign an interpretation to “when tomorrow did Susie learn” (see 2b). This is due to the tense clash between “tomorrow” and “did.” Therefore, to test the effect of implicit prosodic packaging, as proposed, it is important to test wh-questions without such tense clashes. This will be investigated in offline questionnaires (Experiment 2).

## Experiment 1

This experiment investigated whether or not the chunking of English wh-questions into visual frames displayed on a computer screen influenced their online processing. It tested two types of unambiguous temporal wh-questions. The location of frame breaks was manipulated in a self-paced reading study.

### Methods

#### Participants

Fourty-eight native English speakers took part in the study as volunteer participants. They were all students at University of Massachusetts Amherst and participated in the study for course credit.

#### Stimuli

Sixteen sets of sentences (a total of 64 sentences) were constructed, as outlined in Table 1 (see Appendix A for test stimuli). Stimuli were created by crossing two factors, SENTENCE TYPE (Main Clause Interpretation vs. Embedded Clause Interpretation) and BREAK LOCATION (Early Break vs. Late Break). The tense of the main and embedded clauses (i.e., future or past) was manipulated so as to make the scope of the temporal wh-phrase unambiguous. In the conditions for Main Clause Interpretation, a temporal wh-phrase (e.g., “when tomorrow,” “which month last year”) must be unambiguously interpreted as part of the main clause or as modifying the main verb, e.g., “learn” [see (a) and (b) in Table 1 for examples with future tensed wh-phrases; see Appendix A for examples with past tensed wh-phrases]. Sentences meeting these conditions used a verb in the main clause whose tense matched the temporal wh-phrase (e.g., “will learn” for a future tensed wh-phrase, “learned” for a past tensed wh-phrase). Such sentences asked, for example, when tomorrow Susie will learn something or which month last year Susie learned something. In the conditions for Embedded Clause Interpretation [see (c) and (d) in Table 1 for examples with a future tensed wh-phrase], a temporal wh-phrase with the future or past tensed adjunct phrase must be unambiguously interpreted as part of the embedded clause or as modifying the embedded verb, e.g., “will make,” “made.” A verb whose tense matched the temporal wh-phrase was used in the embedded clause of the sentences to meet these conditions. Such sentences were interpreted as asking, for example, when tomorrow Bill will make an important phone call or during which month last year Bill made an important phone call. The stimuli were presented to readers in two different ways. For the Early Break conditions, a frame break was inserted immediately after a temporal wh-phrase, e.g., “when tomorrow” [see (a) and (c) in Table 1]. For the Late Break conditions, a frame break was inserted immediately after the main verb, e.g., “learn” [see (b) and (d) in Table 1]. Comprehension questions asking about the content of the stimulus sentences were also created. For example, for the first two example stimuli in Table 1 (a, b), “Who made a phone call?”, and two options for the answer of the question, “Susie” or “Bill,” were constructed (All participants answered more than 80% of the questions correctly). Eighty-two filler sentences varying in syntactic structure were added to the stimulus set.

**Table 1:** **Example sentence stimuli for Experiment 1**.

**Condition**
**a. Main Clause Interpretation, Early Break** When tomorrow/will Susie learn that Bill made an important phone call?
**b. Main Clause Interpretation, Late Break** When tomorrow will Susie learn/that Bill made an important phone call?
**c. Embedded Clause Interpretation, Early Break** When tomorrow/did Susie learn that Bill will make an important phone call?
**d. Embedded Clause Interpretation, Late Break** When tomorrow did Susie learn/that Bill will make an important phone call?

Slashes indicate presentation regions (or frame breaks) in the self-paced reading experiment.

#### Procedures

A moving window self-paced reading technique was used. Participants were asked to read each sentence that appeared on the computer screen frame by frame, as presentenced in Table 1. They were instructed to read each frame of each sentence at their own pace. They were also asked to read the sentences as quickly as possible without sacrificing their comprehension. After each sentence, a comprehension question with two options for its answer appeared in the middle of a computer screen. The question remained on the computer screen until participants chose one of the answer options. Stimuli were counterbalanced into four lists (using Latin Square) and participants were randomly assigned to each list of stimuli.

Data were analyzed in the following way. A repeated measures 2 × 2 ANOVA with SENTENCE TYPE (Main Clause Interpretation vs. Embedded Clause Interpretation) and BREAK LOCATION (Early Break vs. Late Break) was carried out for total reading times, first frame (Region 1), and second frame (Region 2). For the data on Regions 1 and 2, a repeated measures 2 × 2 × 2 ANOVA with SENTENCE TYPE, BREAK LOCATION, and REGION (Region 1 vs. 2) was performed. All statistical analyses were conducted with an error term against participants (*F*_1_) and with an error term against items (*F*_2_). Follow-up comparisons were made when needed. Effects that approached either significance (*p* ≤ 0.05) or marginal significance (*p* ≤ 0.10) are reported in the Results Section.

### Results

Table 2 presents the results of the experiment. For total reading times, a repeated measures 2 × 2 ANOVA with SENTENCE TYPE and BREAK LOCATION showed a significant (or marginally significant) effect of SENTENCE TYPE [*F*_1(1, 47)_ = 8.44, *p* < 0.01, *F*_2(1, 15)_ = 3.02, *p* = 0.10]. No significant effect of BREAK LOCATION or interaction of SENTENCE TYPE and BREAK LOCATION was found (*F*'s ≤ 1). The significant effect of SENTENCE TYPE suggests that sentences with a main clause interpretation (a, b) were, on average, read faster than those with an embedded clause interpretation (c, d) (Main Clause Interpretation vs. Embedded Clause Interpretation: 5673 vs. 6122 ms).

**Table 2:** **Results of Experiment 1**.

**Condition**	**Region 1**	**Region 2**	**Total reading time**
a. Main Clause Interpretation, Early Break	1351 (528)	4343 (1911)	5694 (2106)
b. Main Clause Interpretation, Late Break	2905 (1133)	2746 (1337)	5651 (2107)
c. Embedded Clause Interpretation, Early Break	1272 (487)	4740 (1982)	6012 (2219)
d. Embedded Clause Interpretation, Late Break	3556 (1435)	2675 (1161)	6231 (1964)

Mean reading times (ms) in each region and mean total reading times (i.e., mean reading times for entire sentences). Numbers in parentheses indicate SD of the mean.

For the data for the two regions, a repeated measures 2 × 2 × 2 ANOVA with SENTENCE TYPE, BREAK LOCATION, and REGION was carried out. The results showed a significant (or marginally significant) effect of SENTENCE TYPE [*F*_1(1, 47)_ = 8.44, *p* < 0.01, *F*_2(1, 15)_ = 3.02, *p* = 0.10], REGION [*F*_1(1, 47)_ = 67.32, *p* < 0.0001, *F*_2(1, 15)_ = 72.38, *p* < 0.0001], BREAK LOCATION × REGION interaction [*F*_1(1, 47)_ = 164.23, *p* < 0.0001, *F*_2(1, 15)_ = 730.40, *p* < 0.0001], and SENTENCE TYPE × BREAK LOCATION × REGION interaction [*F*_1(1, 47)_ = 16.10, *p* < 0.001, *F*_2(1, 15)_ = 13.81, *p* < 0.01]. The three way significant interaction was resolved by SENTENCE TYPE. For the Main Clause Interpretation, a repeated measures 2 × 2 ANOVA with BREAK LOCATION and REGION showed a significant effect of REGION [*F*_1(1, 47)_ = 59.85, *p* < 0.0001, *F*_2(1, 15)_ = 59.67, *p* < 0.0001] and BREAK LOCATION × REGION interaction [*F*_1(1, 47)_ = 124.24, *p* < 0.0001, *F*_2(1, 15)_ = 291.47, *p* < 0.0001]. For the Embedded Clause Interpretation, significant effect of REGION [*F*_1(1, 47)_ = 51.79, *p* < 0.0001, *F*_2(1, 15)_ = 46.83, *p* < 0.0001] and BREAK LOCATION × REGION interaction [*F*_1(1, 47)_ = 139.11, *p* < 0.0001, *F*_2(1, 15)_ = 334.91, *p* < 0.0001] were found. The significant effect of REGION found for both SENTENCE TYPEs (Main Clause and Embedded Clause Interpretations) comes from Region 1 of the Early Break conditions taking less time to read than the Late Break conditions. Note that the sentences for the Early Break conditions had fewer phrases in Region 1 than the sentences for the Late Break conditions, e.g., “When tomorrow,” “When last month,” compared to Region 1 of the Late Break conditions, which had the entire main clause, e.g., “When tomorrow will/did Susie learn.” The significant interaction between BREAK LOCATION and REGION for both SENTENCE TYPEs was due to the inverse relation in reading times between two regions (Regions 1 and 2) in the Early and Late Break conditions. That is, for both Main Clause and Embedded Interpretations, the Early Break conditions had shorter reading times for Region 1 than Region 2, whereas the Late Break conditions had shorter reading times for Region 2 than Region 1. This is probably explained by the trade-off that occurred in two regions. If we pay the price earlier, then it is less costly later, and vice versa.

### Discussion

The results of Experiment 1 are consistent with the current proposal that frame breaks encourage readers to insert implicit prosodic boundaries where they appear in self-paced reading. On this account, when a frame coincides with a prosodic unit in which the interpretation of part of a sentence occurs (e.g., a clause), the frame is read faster than when it does not. The Late Break conditions [i.e., sentences in (b, d) in Table 1] in Experiment 1 were a crucial test case for this proposal. The first frame (or Region 1) in sentences with Main Clause Interpretation with Late Break (b) allowed a wh-phrase to be interpreted within the first frame. In this condition, a future or past tensed wh-fragment (e.g., “when tomorrow,” “which month last year”) matched the future or past tense of the main clause. In contrast, the wh-phrase in sentences with Embedded Clause Interpretation with Late Break (d) could not be interpreted as part of the first frame. In those sentences, the readers had to wait until they encountered the second frame in order to interpret the wh-fragment. As described here, for the Late Break conditions, the first frame (or Region 1) of the sentences with Main Clause Interpretation (2905 ms) were read faster than that of the sentences with Embedded Clause Interpretation (3556 ms).

Another function of a frame break might be to signal that the upcoming element should not be interpreted together with the element that appears before the frame break. In the present study, the insertion of a frame break after e.g., “when tomorrow,” “which month last year” [Early Break; see (c) in Table 1], might have helped the readers to process the sentences with an embedded clause interpretation, when compared with the Late Break condition [see (d) in Table 1]. Although the reading times in two separate regions of the sentences were not informative, the total reading times for the sentences with Embedded Clause Interpretation support the view that having an early break in those sentences might have facilitated their processing. However, such a view is not likely to be correct based on the present results. As described in the Results Section, a repeated measures 2 × 2 ANOVA for the total reading times showed no significant interaction between SENTENCE TYPE or BREAK LOCATION. Furthermore, for the total reading times, no significant difference was found in a pairwise comparison between the two Break Location conditions of the Embedded Clause Interpretation (*F*'s < 1).

Besides the results discussed above, it should be mentioned that sentences with a main clause interpretation were, on average, read faster than those with an embedded clause interpretation. The significant effect of SENTENCE TYPE in total reading times supports this [see (a) and (b) vs. (c) and (d) in Table 2: 5673 vs. 6122 ms]. This effect can be explained by Active Filler Strategy or Minimal Chain Principle, as mentioned earlier, i.e., the sentence processor inserting a gap (or a trace) at the earliest syntactically legal position as soon as a filler (or a wh-phrase) is encountered or its producing the minimal number of empty categories (or traces). In addition, when a frame break appeared at a place which did not chunk the temporal wh-phrase and the main clause in the same prosodic unit [i.e., Early Break conditions; see (a) and (c) in Table 1], sentences with a main clause interpretation took shorter times in Region 2 (4343 ms) than those with an embedded clause interpretation (4740 ms).

It should also be noted that there may be at least two alternative explanations for the results discussed above. It may be the case the driving force for the results is not implicit prosody or packaging of the materials that can be interpreted together in the same prosodic unit, as proposed. Note that there was no clear negative effect of having e.g., “when tomorrow” and “when last month” in one frame, i.e., without putting the temporal wh-phase and a tense matched clause in the same frame break for interpretation. Rather, the results might be attributed to the syntactic structure of the test sentences. It may be that the sentences were processed faster when the temporal wh-phrase and its trace (see 4a below) appeared in the same frame break, as compared to when it did not (see 4b below):

(4) Slashes indicate frame breaks in the self-paced reading study.
a. Main Clause Interpretation, Late Break
When tomorrow_i_ will Susie learn t_i_/that Bill made an important phone call?
b. Embedded Clause Interpretation, Late Break
When tomorrow_j_ did Susie learn/that Bill will make an important phone call t_j_?

In addition, there is always a tense clash between the temporal wh-phrase (e.g., future tense) and the tense of the main clause (e.g., past tense) for the conditions with the Embedded Clause Interpretation (see 4b above and 5 below), regardless of the location of the frame breaks. This might have resulted in slower reading times for the sentences with the Embedded Clause Interpretation.

(5) Embedded Clause Interpretation, Early Break
When tomorrow_j_/did Susie learn that Bill will make an important phone call t_j_?

The present experiment supports the hypothesis that frame breaks can be treated as implicit prosodic boundaries, which, in turn, influence the readers' interpretation of the temporal wh-phrases. However, as noted above, some alternative accounts cannot be ruled out. In the following experiment, scopally ambiguous wh-questions were tested for their interpretation preference in offline questionnaires. It investigated whether or not line breaks in text can invite implicit prosodic boundaries and help the reader assign an interpretation to the ambiguous wh-phrase. Since the ambiguous temporal wh-questions were tested, the potential difficulty triggered by the tense clash shown in (4b) and (5) above was avoided. In addition, by having one of the questionnaire versions show the entire sentence in one line (see below), the possible syntactic account was likely to be circumvented due to the presence of potential (invisible) traces or empty categories in one line.

## Experiment 2

Experiment 2 tested whether or not line breaks in offline questionnaires create a processing basis similar to those observed in frame breaks in the self-paced reading study in Experiment 1. Scopally ambiguous temporal wh-questions were tested. As described below, two versions of an offline questionnaire were created in order to manipulate the location of line breaks. One version looked like an ordinary offline questionnaire and the other version was formatted with two columns as in newspapers and some journal articles.

### Methods

#### Participants

A total of 80 students participated in the study. The recruiting procedures were the same as in Experiment 1. None of the participants took part in Experiment 1.

#### Stimuli

The stimuli tested in this study were scopally ambiguous wh-questions, as shown in Table 3. The temporal wh-fragments were interpreted as part of either the main clause or the embedded clause (see Appendix B for test stimuli). A total of 16 sentences were constructed. Half of the sentences were constructed using the future tense, e.g., “When next year will the principal announce that a computer company will make a donation?” The other half of the sentences used the past tense, e.g., “When yesterday did Mike proclaim that a UFO was seen in western Arizona?” As presented in Table 3, two versions of the questionnaire were created: Line Break vs. No Line Break. In the Line Break version, a line break was always inserted after the main verb, e.g., “learn,” “proclaim.” That is, the entire sentence took two lines. In the No Line Break version, the entire sentence appeared in one line without a line break inserted. Following each sentence, there was a comprehension question asking which interpretation the reader assigned to the sentence, a main clause interpretation or an embedded clause interpretation (see Table 3). An additional 44 sentences were included as fillers. The filler sentences varied in their syntactic structures.

**Table 3:** **Example sentence stimuli for Experiment 2**.

**Line Break Version**
**When tomorrow will Susie learn that Bill will make an important phone call?** The sentence asks: _ when Susie will learn something _ when Bill will make an important phone call
**No Line Break Version**
**When tomorrow will Susie learn that Bill will make an important phone call?** The sentence asks: _ when Susie will learn something _ when Bill will make an important phone call

#### Procedures

The experiment used a between-participants and within-items design. Half of the participants were randomly assigned to the Line Break version of the questionnaire and the other half to the No Line Break version of the questionnaire. Participants were given a questionnaire and asked to fill it out as quickly as they could without sacrificing their comprehension.

For data analyses, the mean percentage of the main clause interpretation chosen for the test sentences was computed for the two versions of the questionnaire. Following this, a one way ANOVA on the factor VERSION (Line Break vs. No Line Break) was carried out to compare the interpretation choice for the test sentences between the two versions of the questionnaire.

### Results

**Figure 1:**
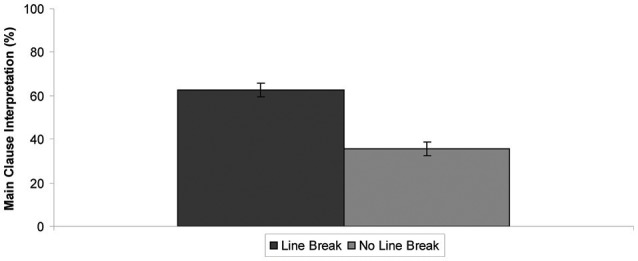
**Results of Experiment 2**. The *x*-axis represents experimental conditions (or questionnaire versions). The dark bar corresponds to the Line Break version of the questionnaires and the light bar to the No Line Break version of the questionnaires. The *y*-axis represents the mean percent of the main clause interpretation chosen for the test sentences. The error bars indicate standard errors of the mean.

Figure 1 presents the results of Experiment 2. As shown in Figure 1, in the case of scopally ambiguous wh-questions, the main clause interpretation was chosen more often than the embedded clause interpretation in the Line Break version (64% preference for the main clause interpretation). This was not true in the No Line Break version (37% preference for the main clause interpretation). The statistical analysis supports this finding. A significant effect of VERSION was found [*F*_1(1, 78)_ = 36.07, *p* < 0.0001, *F*_2(1, 15)_ = 55.24, *p* < 0.0001]. There was no significant difference between the sentences with the future vs. past tense in both Line Break and No Line Break versions (*F*'s < 1).

### Discussion

The results of the present experiment demonstrated that the line breaks that appeared in the offline questionnaire study resulted in a bias toward the main clause interpretation for sentences that were ambiguous with respect to the interpretation of the temporal wh-fragments, e.g., “when tomorrow,” “which month last year” (64% preference for the main clause interpretation). Such an effect can be explained by assuming that an implicit prosodic boundary was inserted immediately after a line break, i.e., after the main verb before the complimentizer “that”; the wh-fragment and the main clause were (implicitly) prosodically packaged together. This implies that the line breaks in the questionnaire study and the frame breaks in the self-reading study behaved alike.

As shown in Figure 1, for scopally ambiguous wh-questions, no clear preference toward the main clause interpretation over the embedded clause interpretation was found (overall 51% preference toward the main clause interpretation). Active Filler Strategy favors the main clause interpretation over the embedded clause interpretation, as the former predicts a trace for the wh-phrase within the main clause, not within the embedded clause. Several factors may be playing a role in the result found in the current study. The tested wh-fragments were all adjuncts (e.g., “when yesterday,” “which month next year”), not arguments (e.g., what, who). The tested sentences were rather complex and long. Also, an offline questionnaire method was used, which may have allowed the participants to read the same sentence more than once.

## General discussion

The aim of the present study was to test whether frame breaks in self-paced reading or line breaks in text invite implicit prosodic boundaries, which, in turn, prosodically package together elements within a sentence. We hypothesized that the processing of a sentence is facilitated when the elements within a sentence that can be interpreted together are placed in the same implicit prosodic unit. Under this hypothesis, breaks in text should not only aid the processing of unambiguous wh-questions but also introduce an interpretation bias for scopally ambiguous wh-questions.

A self-paced reading study (Experiment 1) showed that placing a temporal wh-phrase and its tense matched main verb in the same implicit prosodic unit via frame breaks resulted in faster reading times. We interpret this result to mean that having the temporal wh-phrase and the tense matched main verb presented in the same frame helped the reader to interpret the materials together by prosodically packaging them. Specifically, the first region (or the first frame) for the Late Break condition for the Main Clause Interpretation was read faster than the corresponding region for the same break condition for the Embedded Clause Interpretation. The data from this experiment also indicated that the following region (i.e., Region 2) took less time to read for the Late Break condition for the Main Clause Interpretation than the same break condition for the Embedded Clause Interpretation. These results in reading times in Regions 1 and 2 resulted in a difference in total reading times for the Late Break condition with the Main Clause Interpretation being read faster than the corresponding break condition with the Embedded Clause Interpretation. Overall, this experiment showed that scopally unambiguous wh-questions are read faster when they are presented with frame breaks that chunk the materials so that they can be interpreted.

Although it was an offline questionnaire study, the results of Experiment 2 also provide support for the proposed hypothesis, i.e., implicit prosodic packaging of the materials that can be processed together has an influence on sentence processing. Experiment 2 tested wh-questions with scopally ambiguous temporal wh-phrases and the interpretation preference for the wh-phrases based on where line breaks occurred. As presented, packaging the wh-phrase and the main clause into the same prosodic unit via line breaks resulted in the participants' choosing a main clause interpretation for the wh-question more often than an embedded clause interpretation.

As mentioned already, the present work relies on a hypothesis that frame breaks in self-paced reading (Experiment 1) and line breaks in text (Experiment 2) induce implicit prosodic boundaries. As noted above, several alternative accounts may be possible. First, the results of Experiment 1 may be explained by the tense clash between the temporal wh-phrase and the tense of the main clause. Experiment 1 showed that it takes longer to read the temporal wh-phrase and the tense mismatched main clause placed in the same frame break, i.e., “When tomorrow did Susie learn,” compared to having the temporal wh-phrase and the tense matched main clause in the same frame break, i.e., “When tomorrow will Susie learn.” Perhaps, the tense clash account can be ruled out, given the results of Experiment 2. Experiment 2 tested scopally ambiguous wh-questions that did not have such a tense clash. The results of Experiment 2 showed that line breaks in text influenced the interpretation of the scopally ambiguous temporal wh-phrase.

Second, the effect of frame breaks in the self-paced reading study in Experiment 1 may be explained by Active Filler Strategy or Minimal Chain Principle. The results of Experiment 1 support the view that the main clause interpretation of the wh-phrase is preferred to the embedded clause interpretation of the wh-phrase. This resulted in faster reading times for the wh-questions with the main clause interpretation than those with the embedded clause interpretation. This result can be accounted for by the Active Filler Strategy or the Minimal Chain Principle, which both require the wh-phrase to be processed as soon as the reader encounters it. However, based on the results of Experiment 2, those principles (i.e., the Active Filler Strategy and the Minimal Chain Principle) may not be the sole explanation for the results obtained for Experiment 1. The offline questionnaires reported in Experiment 2 showed that there was no particular bias toward the main clause interpretation for the scopally ambiguous temporal wh-questions (51% for the choice of the main clause interpretation across all sentences across the two versions of the questionnaire). Of course, this observation might be due to the difference in the given tasks. Whereas a self-paced reading study might encourage the incremental processing of sentences, offline questionnaires may not ensure such a premise. It is important that in the future an online version of Experiment 2 be conducted, so that we can compare the results of the two different tasks (See Koizumi and Bradley, 2007 for their results on readers' interpretation for English scopally ambiguous sentences with “negation” and “because,” which used the reading task similar to the self-paced reading studies reported in this work).

Third, it might be argued that the results obtained are better explained by some syntactic effect. For example, frame breaks or line breaks may insert traces or other empty categories for the temporal wh-phrase during online processing. Since traces or empty categories are invisible in text, this explanation is not easy to rule out. Also, as proposed by many researchers, it is often the case that syntactic boundaries and prosodic boundaries coincide (Selkirk and Tateishi, 1991; see also Deguichi and Kitagawa, 2002; Ishihara, 2002, and the references therein). This makes it difficult to argue for an independent effect of implicit prosody in reading text.

Even though a syntactic account for the reported results is not easy to rule out, we have a considerable amount of evidence suggesting that implicit prosody plays an important role in online processing of sentences (see the Introduction Section). Based on previous work, it is probably not far-fetched to argue that frame breaks in self-paced reading or line breaks in text induce implicit prosodic boundaries during reading. There is eye-tracking evidence that suggests punctuation marks (i.e., comma, period) in English text likely trigger an implicit prosodic boundary (Hirotani et al., 2006; For German evidence regarding a comma effect inducing an implicit prosodic boundary, see Steinhauer and Friederici, 2001). It is probably natural to assume that the reader hears his or her voice while reading (e.g., Bader, 1998; Fodor, 2002) and stops when he or she encounters frame breaks or line breaks, just like when he or she hits a comma or a period during reading. As mentioned already, it is important to gather more evidence in the future that frame breaks or line breaks induce implicit prosodic boundaries that help or hinder the reader's processing of sentences on-line.

The present study may have important practical implications. If frame breaks invite prosodic boundaries in self-paced reading, researchers must be careful in setting up of their reading experiments. If line breaks invite implicit prosodic boundaries, as hypothesized in this study, writers are encouraged not to enter line breaks at random places, or, at least, not to place them in locations that might hinder readers' sentence interpretation. This may not be easy to achieve, given the cost of printing and other factors. However, the results of Experiment 2 imply that those used to reading papers with two columns may have been influenced by the location of line breaks. It is probably a good idea to keep this in mind when writing sentences. Related to these results, other researchers have demonstrated an effect of spacing in printed text on its readability using a computer program (see Bever et al., 1991). Unlike the present study, the focus of that work was syntax, not prosody. Still, the day may come when the effect of implicit prosody in written text will be more systematically investigated.

It will also be important to test the effect of prosodic packaging and line breaks with different types of sentences. Identifying sentence types that are subject to the effect more than others is critical. Also, in need of investigation is whether varying the location of frame breaks and line breaks within a sentence changes their effectiveness. Finally, it is crucial to assess whether or not the effect of frame breaks and line breaks is something stable that always needs to be taken into account during online sentence processing.

## Conclusion

This study presented two experiments whose results support the hypothesis that breaks in text induce implicit prosodic boundaries during reading. Furthermore, they demonstrated that prosodic packaging of parts of a sentence together created by the insertion of implicit prosodic boundaries influences the processing of the sentence. Specifically, reading times of wh-questions in a self-paced reading study were shorter when the wh-questions were presented with frame breaks that packaged parts of the wh-questions that could be interpreted together than when they were not. In offline questionnaires, the location of line breaks introduced a bias in the interpretation of ambiguous wh-questions. A wh-phrase was interpreted as part of a main clause more often when a line break was inserted after the main clause than when it was not.

## Author contributions

MH carried out all aspects of the reported studies. Both JT and NS provided critical comments to the reported work and wrote the manuscript with MH.

## Funding

This study was partly supported by JSPS Grant-in-Aid for Scientific Research (A) “An investigation of the automatization process in language processing with respect to the development of foreign language proficiency” (PI: Hirokazu Yokokawa, No. 21242013).

### Conflict of interest statement

The authors declare that the research was conducted in the absence of any commercial or financial relationships that could be construed as a potential conflict of interest.

## Appendices

### Appendix A

Experimental stimuli for Experiment 1 are provided below. The experiment had a total of 16 items; half used a future tensed wh-phrase (1)–(8), and the other half used a past tensed wh-phrase (9)–(16). In the first two conditions (see 1a and 1b), the wh-phrase unambiguously attached to the main verb (Main Clause Interpretation), and in the second two conditions (see 1c and 1d), the wh-phrase unambiguously attached to the embedded verb (Embedded Clause Interpretation). In order to control the attachment site of the wh-phrase, the tense of the main and embedded verbs was manipulated. A frame break was inserted after the wh-phrase in the first and third conditions (Early Break; see 1a and 1c) and after the main verb in the second and fourth conditions (Late Break; see 1b and 1d).

1
  - a. When tomorrow
  - will Sue learn that Dan made an important phone call?
  - b. When tomorrow will Sue learn
  - that Dan made an important phone call?
  - c. When tomorrow
  - did Sue learn that Dan will make an important phone call?
  - d. When tomorrow did Sue learn
  - that Dan will make an important phone call?
2. When next month will/did the Dean proclaim that Joe's probation started/will start?
3. When next week will/did Rachel say that Tom visited/will visit Grenada?
4. Which month next year will/did the principal announce the teacher made/will make a donation?
5. When next spring will/did Ann reveal to Mother that Kim had/will have a baby?
6. Which day next week will/did the lawyer claim that the client left/will leave?
7. Which week next month will/did Pam inform her husband that the gift arrived/will arrive?
8. Which month next year will/did the mechanic report that the tires needed/will need to be changed?
9. When yesterday did/will Mike proclaim that a UFO will be seen/was seen in El Paso?
10. When last month did/will the coach say that the athletes will have/had a match?
11. When last week did/will Cathy learn that Greg will see/saw old temples in Japan?
12. When last winter did/will the president announce that a new law will be enacted/was enacted?
13. When last year did/will Ellen guess that Lisa will get/got married over vacation?
14. When last spring did/will the secretary claim that the department will resume/resumed old procedures?
15. Which day last month did/will Jennifer report to her advisor that Bill will start/started working?
16. Which month last year did/will the mother tell the judge that the kids will be/were videotaped?

#### Appendix B

Experimental stimuli for Experiment 2 are provided below. All sentences are ambiguous with respect to the scope of their wh-phrase, the wh-phrase attaching either to the main clause or the embedded clause. Sentences (1)–(8) used future tense for both main and embedded clauses, whereas sentences (9)–(16) used past tense. As illustrated in Table 3, the Line Break questionnaire had a line break after the main verb. The No Line Break questionnaire had no line break throughout the entire sentence.

1. When tomorrow will Sue learn that Bill will have knee surgery?
2. When next month will the Dean proclaim that Joe's probation will start?
3. When next week will Rachel say that Tom will visit Grenada by train?
4. When next year will the principal announce a computer company will make a donation?
5. When next spring will Ann reveal to Mother that Kim will adopt a baby?
6. Which day next year will the lawyer claim that the criminal will be dismissed?
7. Which day next week will Pam inform her husband that the gift will arrive?
8. Which month next year will the mechanic report that the car will need its tires changed?
9. When yesterday did Mike proclaim that a UFO was seen in western Arizona?
10. When last month did the coach say that the athletes had a match?
11. When last week did Cathy learn that Greg saw old temples in Japan?
12. When last winter did the president announce that a new law was enacted?
13. When last year did Ellen guess that Lisa got married in France?
14. When last fall did the secretary claim that the department resumed the rule?
15. Which day last month did Jen report to her boss that Matt started working?
16. Which month last year did mom tell the judge that the kids were taped?
